# Nuclear genome-derived circular RNA circPUM1 localizes in mitochondria and regulates oxidative phosphorylation in esophageal squamous cell carcinoma

**DOI:** 10.1038/s41392-021-00865-0

**Published:** 2022-02-14

**Authors:** Wei Gong, Jiancheng Xu, Yan Wang, Qingjie Min, Xu Chen, Weimin Zhang, Jie Chen, Qimin Zhan

**Affiliations:** 1 Key Laboratory of Carcinogenesis and Translational Research (Ministry of Education/Beijing), Laboratory of Molecular Oncology, Peking University Cancer Hospital & Institute, 100142 Beijing, China; 2 Institute of Systems Biomedicine, School of Basic Medical Sciences, Peking University Health Science Center, 100191 Beijing, China; 3 Peking University International Cancer Institute, 100191 Beijing, China; 4 Shenzhen Bay Laboratory, Shenzhen, 518132 China; 5 Research Unit of Molecular Cancer Research, Chinese Academy of Medical Sciences, 100021 Beijing, China

**Keywords:** Non-coding RNAs, Cancer microenvironment

## Abstract

Circular RNAs (circRNAs) were shown to play an important role in the occurrence and progression of tumors. However, the functions of nuclear genome-derived circRNAs localized in mitochondria of tumor cells remain largely elusive. Here, we report that circPUM1, a circular RNA derived from back-splicing of pre-mRNAs of nuclear genome PUM1, localizes in mitochondria. The expression level of circPUM1 is positively correlated with HIF1α accumulation under CoCl_2_-induced intracellular hypoxic-like condition in esophageal squamous cell carcinoma (ESCC) cell lines. Importantly, circPUM1 acts as a scaffold for the interaction between UQCRC1 and UQCRC2 in ESCC cell lines. Knock-down of circPUM1 would result in lower intracellular oxygen concentration, downregulated oxidative phosphorylation, decrease of mitochondrial membrane potential, increase of ROS generation and shrinking of mitochondria, respectively. CircPUM1 depletion induces dysfunction of the mitochondrial complex III and the cleavage of caspase3 spontaneously. Interestingly, disruption of circPUM1 led to pyroptosis that initiates the cell death of ESCC cell lines. Therefore, we conclude that circPUM1 plays a critical role in maintaining the stability of mitochondrial complex III to enhance oxidative phosphorylation for ATP production of ESCC cells and moreover propose that ESCC cells exploit circPUM1 during cell adaptation.

## Introduction

Esophageal cancer is one of the most common malignant tumors in the world, ranking sixth among cancer-related deaths.^1^ Esophageal cancer mainly includes two pathological types: esophageal squamous cell carcinoma (ESCC) and esophageal adenocarcinoma (EAC). Nearly 90% of the world’s patients with ESCC are in China. The five-year survival rate for patients is still only 15–25%. Currently, there is still a lack of effective targeted therapeutic drugs.^2,3^

A number of investigations have reported that the energy metabolism of tumor cells is different from normal cells.^4^ Even in the case of sufficient oxygen, tumor cells preferentially provide energy by means of glycolysis, which is known as the Warburg effect.^5^ In addition, rapidly proliferating tumor cells are often considered to be in a hypoxic state. Hypoxia induction factor1α (HIF1α) is the core regulator in tumor cells following hypoxia and has long been connected with the Warburg effect.^6^ As a transcription factor, HIF1α activates the expression of glycolytic genes and promotes glycolysis. It has been reported that typical HIF1α target genes include VEGF, GLUT1 and EPO and so on. The signal pathways of HIF1α include PI3K-Akt-HIF1α pathway, SENP1-HIF1α pathway and MAPK-HIF1α pathway.^7–9^ Moreover, a number of studies demonstrated that oxidative phosphorylation was not damaged or even enhanced in some types of tumor cells, such as leukemias and endometrial carcinoma.^10^ Enhanced oxidative phosphorylation could further lead to hypoxia in tumor cells, but the mechanism for increased oxidative phosphorylation in tumor cells is still unclear. So far, there are few reports on whether circRNAs are involved in the contribution of oxidative phosphorylation of tumor cells.

CircRNAs are a class of covalently closed cyclic RNA molecules, most of which are resistant to RNase R digestion and are generally produced by the back-splicing of linear pre-mRNA.^11,12^ CircRNAs participate in a variety of biological processes including cell proliferation, differentiation and metabolism through acting as a microRNA sponge and interacting with proteins.^13,14^ Recently, the correlation between circRNAs and tumors has been gradually elucidated.^15^ It has been shown that circRNAs are closely related to the proliferation, invasion and migration of tumor cells and that dysregulation of circRNAs are related with many kinds of cancers, such as, breast cancer, colorectal cancer, liver cancer, ESCC and so on.^16–19^ However, the function of circRNAs in occurrence and development of tumors need to be further studied.

In this study, we investigated how HIF1α-related circRNA is involved in the development of ESCC using the cobalt chloride-induced chemical hypoxic model. When cobalt chloride inhibited the degradation of HIF1α, we found that the expression level of circPUM1 derived from *PUM1* greatly increased. Interestingly, circPUM1 could regulate the oxidative phosphorylation of mitochondria by interacting with UQCRC2 in mitochondrial complex III. Importantly, circPUM1 could promote the proliferation of ESCC in vivo and in vitro by inhibiting tumors pyroptosis, which provide novel insights into circRNA-regulated oxidative phosphorylation in mitochondria during tumor malignant development.

## Results

### High-throughput identification of circPUM1 in CoCl_2_-induced intracellular hypoxic-like microenvironment model

**Fig. 1:**
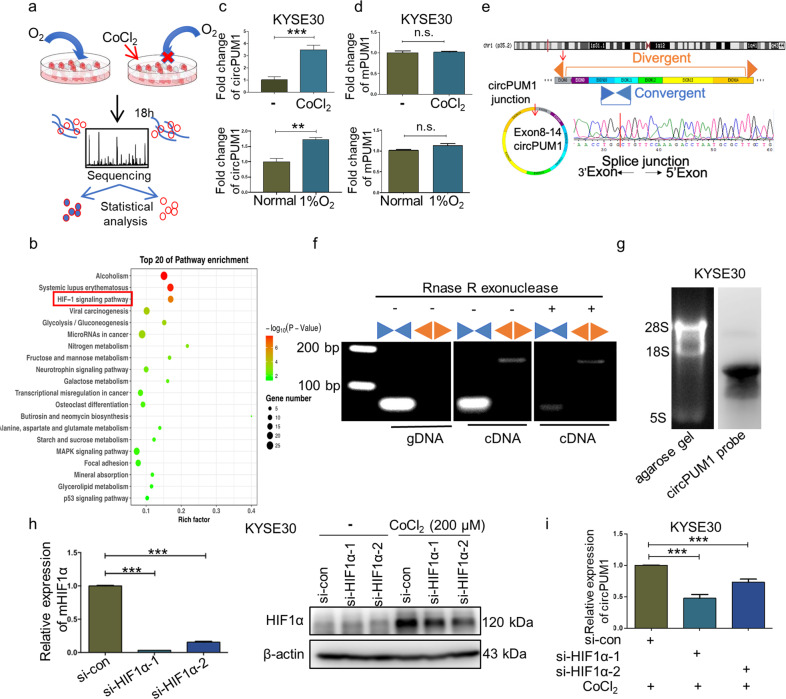
High-throughput identification of circPUM1 in CoCl_2_-induced intracellular hypoxic-like microenvironment model. **a** Intracellular hypoxic-like microenvironment model creation and sequencing strategy. **b** Pathway enrichment analysis of differentially expressed genes. **c** RT-qPCR assay to determine circPUM1 expression level after cells were treated with 200 μM CoCl_2_ for18 h or incubated in 1% oxygen for 24 h. **d** RT-qPCR assay to determine PUM1 mRNA expression level after cells were treated with 200 μM CoCl_2_ for 18 h or incubated in 1% oxygen for 24 h. **e** Sequencing analysis of head-to-tail splicing junction in circPUM1. **f** Divergent and convergent primers were used to amplify circPUM1 and mPUM1 from gDNA, cDNA without RNase R treatment before RT-PCR and cDNA with RNase R treatment before RT-PCR in KYSE30 cell, respectively. **g** Northern blotting analysis of circPUM1 in KYSE30 cell. **h** RT-qPCR and western blot analyses for the knockdown efficiency of si-HIF1α in KYSE30 cell. **i** RT-qPCR assay for circPUM1 expression following the depletion of HIF1α and the treatment with 200 μM CoCl_2_ for 18 h in KYSE30 cell

To explore the roles of intracellular hypoxic-like microenvironment-related circRNAs in tumor development, we constructed a CoCl_2_-induced intracellular hypoxic-like microenvironment model in esophageal squamous cell carcinoma (ESCC) cell line KYSE30 (Fig. 1a). CoCl_2_, a well-known hypoxia-mimicking reagent, is able to replace the ferrous ions located in the catalytic site of proline hydroxylase and aspartate hydroxylase, thus suppressing their ability of degrading HIF1α. As a consequence, CoCl_2_ treatment in cells could elicit an elevated expression of HIF1α, which mimics the intracellular bioenergetic alterations of tumor hypoxia.^20^ We first examined the expression of HIF1α by western blot at different time points in KYSE30 treated by 200 μM CoCl_2_ and found that the expression of HIF1α was elevated in a time-dependent pattern. HIF1α expression increased at 6 h and peaked at 18 h following treatment of CoCl_2_ (Fig. 1a, Supplementary Fig. 1a). Based on these observations, we performed the whole transcriptome sequencing to identify circRNAs in KYSE30 cells treated with CoCl_2_ for 18 h.

Through the sequencing analysis, we identified 179 differentially expressed circRNAs in ESCC intracellular hypoxia model, of which 106 were upregulated and 73 were downregulated (fold-change >2, *p* < 0.05). Additionally, we performed (Gene Ontology) GO analysis (Supplementary Fig. 1b) and pathway analysis of the differentially expressed mRNAs to identify their enriched biological function categories. As Fig. 1b shown, in 18 h CoCl_2_ -treated group, the differentially expressed mRNAs were mainly enriched in cell hypoxia category, which further confirmed the validity of the KYSE30 intracellular hypoxia model. In order to screen out the circRNAs for use in subsequent functional research, we chose potential target circRNAs for further screening from significantly upregulated circRNAs in the ESCC intracellular hypoxia model. Next, RT-qPCR assay was performed to verify above results, as depicted in Fig. 1c and Supplementary Fig. 1c, although circPUM1 as well as circSLC8A1 and circZNF292 were significantly upregulated in the intracellular hypoxia model, their linear form, except PUM1, had also been changed significantly. Additionally, we incubated KYSE30 cells in 1% oxygen for 24 h to test the expression level of circPUM1 under hypoxic condition. Upon exposure to low concentration of oxygen, HIF1α protein expression was substantially enhanced (Supplementary Fig. 1d). Consistently, circPUM1 was significantly upregulated after the depletion of oxygen (Fig. 1c), while neither CoCl_2_ treatment nor low oxygen changed the expression of *PUM1* mRNA (Fig. 1d). Given that, we chose circPUM1 for subsequent further investigations.

CircPUM1 is generated from the *PUM1* gene located on human chromosome 1. According to the annotation of circPUM1 in the circBase, it contains 7 exons ranging from the 8^th^ to 14^th^ exon of *PUM1* gene (total 1168 bp).^21^ Firstly, we sequenced the head-to-tail splicing junction of circPUM1 derived from KYSE30, which was identical to the annotation in the circBase (hsa_circ_0011240) (Fig. 1e). Next, we applied PCR assays to detect the head-to-tail splicing of endogenous circPUM1 with convergent and divergent primers. As shown in Fig. 1f, the divergent primers of circPUM1 amplified a PCR product consistent with its circular form. Moreover, total RNA treated with RNase R, which could degrade linear RNA, before PCR amplification demonstrated that circPUM1 is resistant to RNase R as expected (Fig. 1f). In addition, northern blot was performed to confirm the existence of circPUM1 (Fig. 1g). To further verify the hypothesis that the elevated circPUM1 expression was associated with HIF1α upregulation induced by CoCl_2_ treatment, we knocked down the HIF1α expression by siRNAs in KYSE30 cells (Fig.1h). The results showed that the expression of circPUM1 decreased significantly following the depletion of HIF1α (Fig. 1i). Collectively, circPUM1 is a bona fide circRNA potentially involved in the CoCl_2_-induced KYSE30 intracellular hypoxia and we chose ESCC as tumor research model for subsequent functional studies of circPUM1.

### circPUM1 enhances the tumorigenicity of ESCC cells in vitro and in vivo

**Fig. 2:**
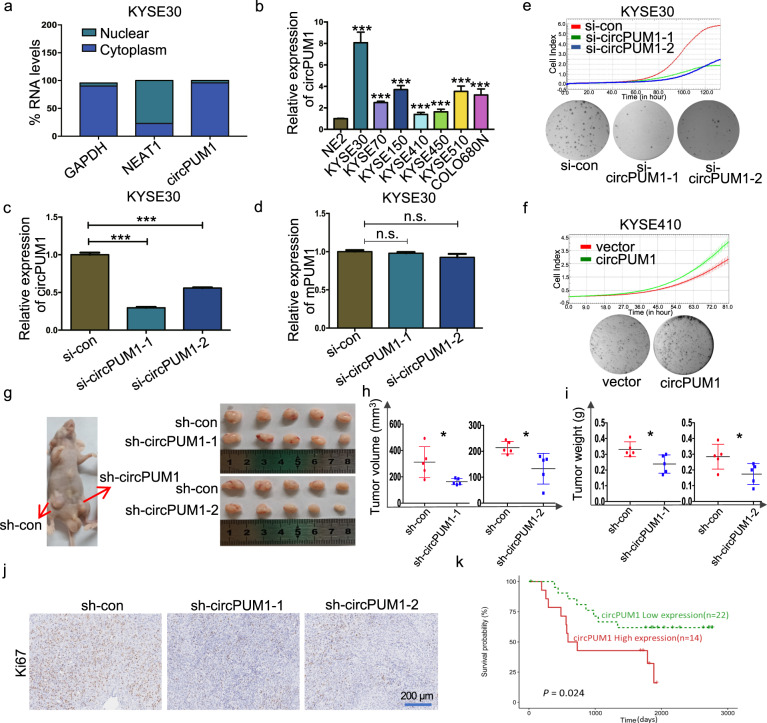
CircPUM1 enhances the tumorigenicity of ESCC cells in vitro and in vivo. **a** Fractionation of KYSE30 cells following by RT-qPCR was used to determine the localization of circPUM1. GAPDH served as the cytoplasmic expression control and NEAT1 severed as the nuclear expression control. **b** RT-qPCR analysis of circPUM1 expression profile in ESCC cell lines that normalized by normal esophageal epithelial cell line NE2. **c** RT-qPCR analysis of circPUM1 after knocking down circPUM1 in KYSE30 cell. **d** RT-qPCR analysis of mPUM1 after knocking down circPUM1 in KYSE30 cell. **e** The growth curve that monitored by the RTCA-MP system and colony formation analyses following the depletion of circPUM1 in KYSE30 cell. **f** The growth curve that monitored by the RTCA-MP system and colony formation analyses following the overexpression of circPUM1 in KYSE410 cell. **g** Macroscopic appearance of the xenograft tumors in nude mice after injection with KYSE30-sh-con (left side on both), KYSE30-sh-circPUM1-1 (right side) (up) and KYSE30-sh-circPUM1-2 (right side) (down). Xenograft tumors were isolated, KYSE30-sh-con (up on both side), KYSE30-sh-circPUM1-1 and KYSE30-sh-circPUM1-2 (down on both side). **h**–**j** Comparison of the growth of KYSE30 xenografts expressing sh-con versus sh-circPUM1 in tumor volume (**h**) and tumor weight (**i**); IHC that with Ki67 antibody staining (**j**), the scale bar is 200 μm. **k** Elevation of circPUM1 in ESCC patients was associated with worse overall survival of ESCC patients

We next examined the subcellular distribution of circPUM1 in KYSE30. Through RT-qPCR assay of total RNAs derived from the nuclear and cytoplasmic fractions respectively, we found that circPUM1 was mainly localized in the cytoplasm (Fig. 2a). To explore the role of circPUM1 in ESCC cells, we evaluated the expression profile of circPUM1 in different ESCC cell lines. It turned out that compared with the normal epithelial cell NE2, circPUM1 was more expressed in ESCC cells, among which KYSE30 cell line and KYSE410 cell line had the highest and lowest expression level of circPUM1 respectively (Fig. 2b). Although circPUM1 has the common exons with PUM1 mRNA, head-to-tail splice junction is absent in circPUM1 host gene. In view of this, we designed siRNAs against back-splicing between exons 8 and 14 of circPUM1. RT-qPCR results showed that siRNAs selectively knockdown of the circPUM1 expression showed no impacts on PUM1 mRNA expression (Fig. 2c, d). Similarly, ectopically expressed circPUM1 in both KYSE30 (Supplementary Fig. 2a) and KYSE410 cells (Supplementary Fig. 2b) did not interfere with endogenous PUM1 mRNA expression (Supplementary Fig. 2c).

We manipulated circPUM1 expression in vitro to explore its role in the regulation of cell growth in ESCC cells. After circPUM1 depletion in KYSE30 cells, reduced cell proliferation and colony formation were observed (Fig. 2e), while circPUM1 overexpression resulted in increased cell proliferation and colony formation in KYSE410 (Fig. 2f) as well as KYSE30 cells (Supplementary Fig. 2d). To evaluate the tumorigenic role of circPUM1 in vivo, circPUM1 was knocked down by short hairpin RNAs (shRNAs) whose core sequence were identical with previous mentioned siRNAs in KYSE30 cells (Supplementary Fig. 2e, f) and the circPUM1 knockdown cells were subcutaneously injected into nude mice. As shown in Fig. 2g, circPUM1 knockdown led to smaller tumors visually, decreased tumor volume (Fig. 2h) as well as tumor weight (Fig. 2i). Additionally, Ki67 immunohistochemical staining in xenograft tumors demonstrated that circPUM1 knockdown cells harbored lower Ki67 expression (Fig. 2j). When analyzing the circRNA expression matrix derived from our unpublished rRNA-depleted transcriptome sequencing data of ESCC cases and their corresponding clinical information, we observed that elevated expression of circPUM1 was associated with worse overall survival of ESCC patients (Fig. 2k). Collectively, circPUM1 promotes ESCC cell growth.

### CircPUM1 interacts with UQCRC2 in mitochondria

**Fig. 3:**
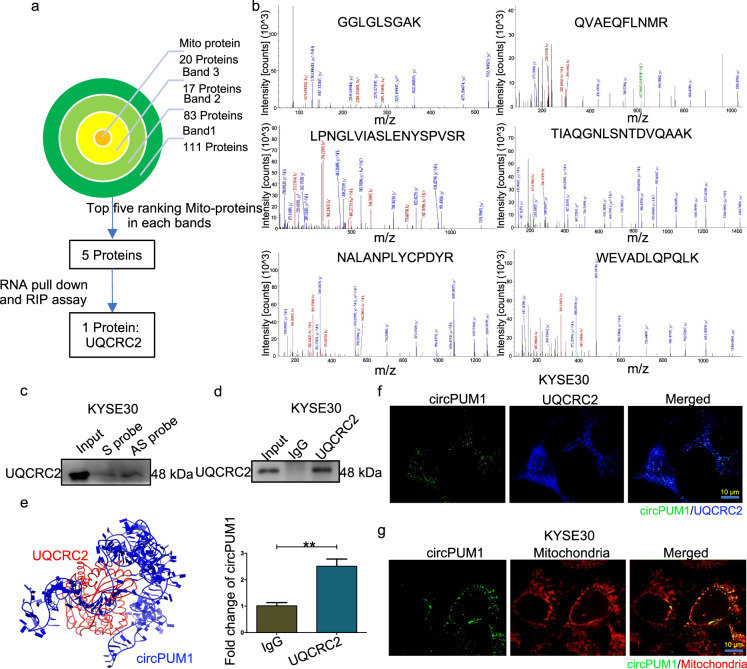
CircPUM1 interacts with UQCRC2 in mitochondria. **a** Analysis pipeline was performed to identify proteins that interact with circPUM1: (1) Screening 20 Mito-proteins from three bands, of which 111 proteins in band 1, 83 proteins in band 2, 17 proteins in band 3; (2) Selecting Mito-proteins that ranked the top five in each band; (3) RNA pulldown-Western Blot and RIP-qPCR analysis confirmed the interaction of UQCRC2 and circPUM1. **b** Mass spectrometry assay depicted the UQCRC2 peptides pulled down by anti-sense circPUM1 probes. **c** Biotin-labeled sense or antisense circPUM1 probes were used for RNA-protein pull-down against KYSE30 cells lysates following western blot analysis of UQCRC2 in precipitated proteins. **d** RIP assay in KYSE30 cells using UQCRC2 antibody. **e** Diagrams showing the interaction of circPUM1 and UQCRC2. **f** Confocal analysis of circPUM1 (green) and UQCRC2 (blue) in KYSE30 cells, the scale bar is 10 μm. **g** FISH detection of circPUM1 (green) and the co-localization with mitochondria (red), the scale bar is 10 μm

To explore the potential molecular mechanism by which circPUM1 implicates in the tumorigenesis of ESCC, we performed RNA pull-down assay to identify circPUM1-interacting proteins using 3’end biotin-tagged anti-sense and sense probes of circPUM1 (Supplementary Fig. 3a). Subsequently, selectively precipitated proteins by circPUM1 anti-sense probe were obtained by silver staining bands recycling and subjected to mass spectrum analysis (Supplementary Fig. 3b). Through the analysis pipeline, we choose UQCRC2, UQCRC1, TOMM40, COA7, and MARCH5 as candidate proteins (Fig. 3a, b) and through RNA pulldown-Western Blot and RIP-qPCR, we demonstrated that UQCRC2 could interact with circPUM1 (Fig. 3c, d). Further, we analyzed the interaction of circPUM1 and UQCRC2 using a computer algorithm on NPDock.^22^ In the process, the secondary structure of circPUM1 was predicted on the RNAfold Web Server. Then the obtained secondary structure was analyzed for further tertiary structure prediction on RNACOmposer.^23^ Furthermore, we acquired the tertiary structure of UQCRC2 from PROTEIN DATA BANK (PDB).^24^ Subsequently, this information was imported into the NPDock. Consistently, as shown in Fig. 3e, the predicted result showed that circPUM1 can interact with UQCRC2. In addition, circPUM1 and UQCRC2 co-localized in the mitochondria of ESCC cells proved by multiple confocal assays (Fig. 3f, g). Taken together, we first identified a nuclear genome-derived circular RNA, circPUM1, which can be located in mitochondria.

### CircPUM1 participates in the regulation of energy metabolism by regulating the mitochondrial oxidative phosphorylation in ESCC cells

**Fig. 4:**
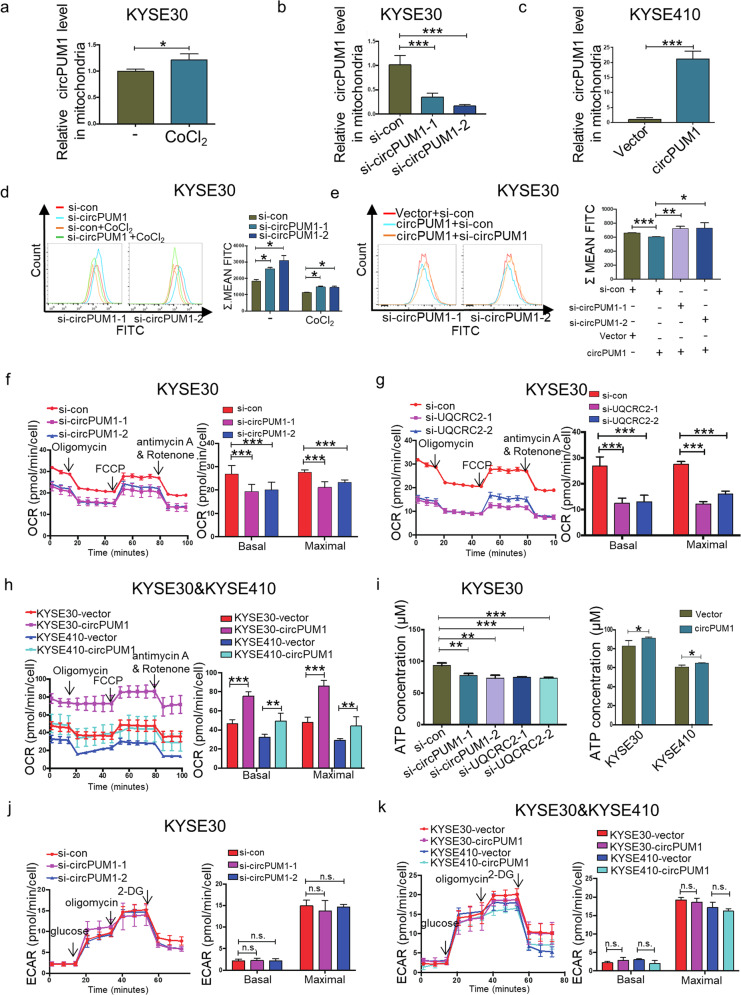
CircPUM1 participates in the mitochondrial oxidative phosphorylation through interacting with UQCRC2. **a** RT-qPCR assay to determine the circPUM1 expression in mitochondria that isolated from KYSE30 cell after treating with 200 μM CoCl_2_ for 18 h. **b** RT-qPCR assay to determine the circPUM1 expression in mitochondria that isolated from KYSE30 cell after knocking down of circPUM1. **c** RT-qPCR assay to determine the circPUM1 expression in mitochondria that isolated from KYSE410 cell after overexpression of circPUM1. **d** Flow cytometry was performed to test hypoxic cells after knocking down circPUM1 with or without CoCl_2_ stimulation in KYSE30 cell. **e** Flow cytometry was performed to test hypoxic cells after knocking down circPUM1 in circPUM1- overexpressed KYSE30 cell. **f** OCR was measured by Seahorse XF assays after knocking down circPUM1 in KYSE30 cell. Basal and maximal OCR levels were at the right side. **g** OCR was measured by Seahorse XF assays after knocking down UQCRC2 in KYSE30 cell. Basal and maximal OCR levels were at the right side. **h** OCR was measured by Seahorse XF assays after overexpressing circPUM1 in both KYSE30 cell and KYSE410 cell. Basal and maximal OCR levels were at the right side. **i** ATP generation assay was performed to test the ATP generation ability in KYSE30 cells (knockdown of circPUM1 or UQCRC2), as well as in KYSE30 and KYSE410 (overexpression of circPUM1). **j** ECAR was measured by Seahorse XF assays after knocking down circPUM1 in KYSE30 cell. Basal and maximal ECAR levels were at the right side. **k** ECAR was measured by Seahorse XF assays after overexpressing circPUM1 in both KYSE30 cell and KYSE410 cell. Basal and maximal ECAR levels were at the right side

The aforementioned circPUM1 subcellular distribution in mitochondria prompted us to explore its role in the regulation of bioenergy metabolism in ESCC. In our CoCl_2_-induced intracellular hypoxic-like microenvironment model in KYSE30 cells, HIF1α, acts as an important oxygen concentration sensor, was protected from degradation and proved to elicit circPUM1 upregulation. Hence, we were enlightened to study whether circPUM1 manipulation would impart effects on the oxygen concentration in ESCC cells. Firstly, we tested the expression level of circPUM1 following treatment of CoCl_2_ in mitochondria. Western blot was performed to test the purity of the isolated mitochondria in KYSE30 cells and KYSE410 cells and VDAC1 was used as the specific marker in mitochondrial fraction, while GAPDH was used as the cytoplasm marker (Supplementary Fig. 4a). The mitochondrial RNAs of CoCl_2_-induced intracellular hypoxic model in KYSE30 cells were subjected to RT-qPCR assays (normalized to mouse long noncoding RNA GADD7, an external reference), and circPUM1 expression was observed to be elevated in mitochondria, which is consistent with its tendency in cytoplasm compared with the normoxic group (Fig. 4a). Besides, circPUM1 knockdown and overexpression assays could respectively lead to circPUM1 downregulation or upregulation in mitochondria as well (Fig. 4b, c). Therefore, both endogenous and exogenous circPUM1 manipulations could affect its expression in mitochondria. Next, we examined the effect of cirPUM1 on oxygen concentration of mitochondria and found that circPUM1 knockdown induced an oxygen concentration decline in ESCC cells with or without CoCl_2_ treatment. Consistently, circPUM1 overexpression led to an oxygen concentration rise in ESCC cells and depletion of the overexpressed circPUM1 could affect such oxygen concentration rise to some extent (Fig. 4d, e). Furthermore, depletion of UQCRC2 (Supplementary Fig. 4b) also resulted in a decrease in oxygen concentration (Supplementary Fig. 4c).

Cancer cells need a large amount of energy to sustain their fast proliferation. Indeed, glycolysis is a preferable manner for tumor cells to obtain energy even in normoxic conditions, referred to as Warburg effect.^25^ However, recent studies challenge the concept with the fact that oxidative phosphorylation (OXPHOS) is also upregulated in certain cancers, including pancreatic ductal adenocarcinoma and breast cancer and so on.^10^ Given the colocalization of circPUM1 and UQCRC2, an important component of complex III in the oxidative respiratory chain, in mitochondria, we evaluated the effects of circPUM1 manipulation on the OXPHOS and glycolysis in KYSE30 cells by Seahorse XF96 Extracellular Flux Analyzers. As shown in Fig. 4f the oxygen consumption rate (OCR) decreased significantly after circPUM1 was deleted in KYSE30 cells. The same results were found in the basal and maximal respiration after circPUM1 depletion in KYSE30 cells (Fig. 4f). Similarly, UQCRC2 depletion could suppress the OCR, basal and maximal respiration significantly in KYSE30 cells (Fig. 4g). We overexpressed the circPUM1 in KYSE30 and KYSE 410 cells and observed that the OCR, basal and maximal respiration were obviously increased after circPUM1 overexpression (Fig. 4h). Moreover, we analyzed the production of ATP and found that circPUM1/UQCRC2 knockdown or circPUM1 overexpression could inhibit or promote the synthesis of ATP, respectively (Fig. 4i). Whereas, neither circPUM1 knockdown nor overexpression could impart effects on the glycolysis in ESCC cells (Fig. 4j, k). Taken together, circPUM1 regulates the mitochondrial energy metabolism through interacting with UQCRC2 in ESCC cells.

### CircPUM1 maintains mitochondrial homeostasis by regulating the mitochondrial complex III assembly

**Fig. 5:**
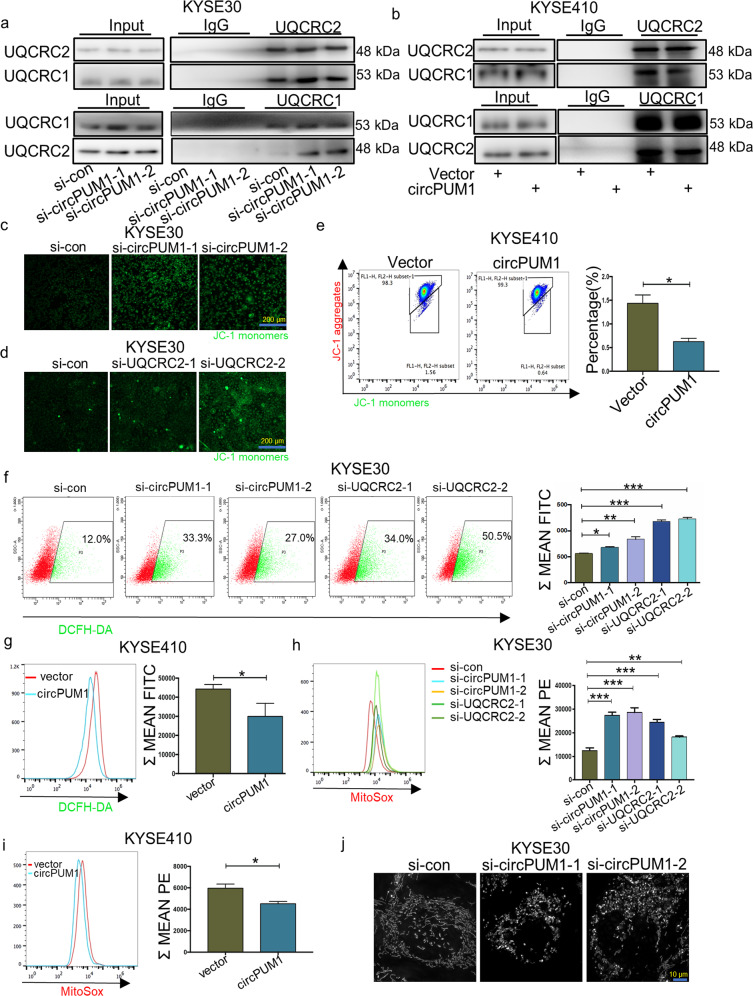
CircPUM1 maintains mitochondrial homeostasis by regulating the mitochondrial complex III assembly. **a** Co-IP analysis for the binding affinity between UQCRC2 and UQCRC1 after knockdown of circPUM1 in KYSE30 cell. **b** Co-IP analysis for the binding affinity between UQCRC2 and UQCRC1 after overexpressing circPUM1 in KYSE410 cell. **c**, **d** Fluorescence microscope was used to observe the change of mitochondrial membrane potential (MMP) after knocking down circPUM1 and UQCRC2 in KYSE30 cell, the scale bar is 200 μm. **e** Flow cytometry was used to analyze the MMP after overexpressing circPUM1 in KYSE410 cells. **f** Flow cytometry was used to analyze the ROS generation after knocking down circPUM1 and UQCRC2 in KYSE30 cells. **g** Flow cytometry was used to analyze the ROS generation after overexpressing circPUM1 in KYSE410 cells. **h** Flow cytometry was used to analyze the MitoSox generation after knocking down circPUM1 and UQCRC2 in KYSE30 cells. **i** Flow cytometry was used to analyze the MitoSox generation after overexpressing circPUM1 in KYSE410 cells. **j** Super Resolution Microscope was used to observe mitochondrial morphology after knocking down circPUM1 in KYSE30 cell

An increasing body of evidence suggests that circRNAs can act as a scaffold in the protein complex assembly.^26^ UQCRC2 interacts with UQCRC1 to form a core dimer in mitochondrial complex III which helps pump hydrion from the mitochondrial matrix to the mitochondrial intermembrane space.^27^ In view of this, we examined the interaction between UQCRC2 and UQCRC1 after circPUM1 depletion or overexpression by Co-IP. As in Fig. 5a, circPUM1 knockdown led to intense interaction between UQCRC2 and UQCRC1. By contrast, circPUM1 overexpression disrupted the interaction between these two proteins (Fig. 5b), suggesting that circPUM1 might serve as a scaffold to regulate the spatial conformation of mitochondrial complex III.

As one of the major hydrogen transmitters in the electron transport chain, mitochondrial complex III is essential for the maintaining of the hydrogen electrochemical gradient across the mitochondrial inner membrane and ATP synthesis in the final step of OXPHOS.^28^ Thus, we examined mitochondrial membrane potential (MMP) alterations after circPUM1 and UQCRC2 manipulation. Clearly, circPUM1 and UQCRC2 depletion substantially decreased the MMP in KYSE30 cells (Fig. 5c, d), while overexpression of circPUM1 enhanced the MMP in KYSE410 cells (Fig. 5e). In addition, overexpression of circPUM1 could rescue the decreased MMP caused by the knockdown UQCRC2 (Supplementary Fig. 5a). Interestingly, we found that decreased MMP was associated with improved ROS. CircPUM1 and UQCRC2 depletion increased the ROS level significantly (Fig. 5f), while circPUM1 overexpression had an opposite effect on ROS (Fig. 5g). Accordingly, overexpression of circPUM1 could decrease the ROS generation induced by knockdown of UQCRC2 (Supplementary Fig. 5b). Furthermore, elevated ROS was seen to mainly come from mitochondrial ROS (Fig. 5h, i and Supplementary Fig. 5c, d). Since mitochondrial dysfunction is usually accompanied by an abnormal mitochondrial morphology, we used super-resolution microscopy to analyze the status of mitochondria in circPUM1 abolished ESCC cells at the sub-organelle level. As depicted in Fig. 5j, the morphology of mitochondria changed from filamentous to fragmented following the depletion of circPUM1, which further illustrates the importance of circPUM1 in maintaining mitochondrial function. In conclusion, circPUM1 maintains mitochondrial homeostasis through providing a scaffold for adjustable mitochondrial complex III assembly.

### CircPUM1 inhibits the pyroptosis in ESCC cells

**Fig. 6:**
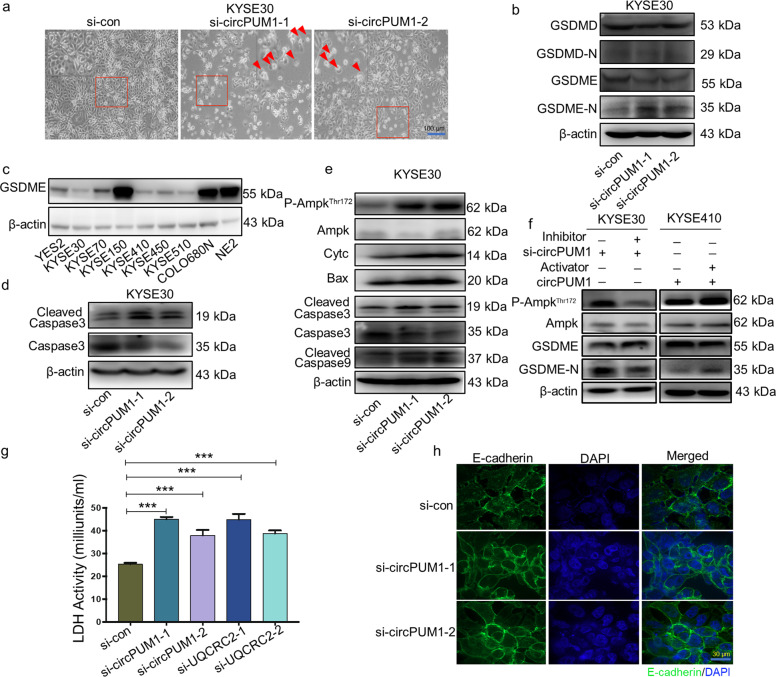
CircPUM1 could inhibit the pyroptosis in ESCC cells. **a** Phase-contrast cell images (arrows, pyroptotic cells) after knocking down circPUM1 in KYSE30 cell, the scale bar is 100 μm. **b** Western blot analysis of knocking down circPUM1-induced GSDME cleavage but not GSDMD cleavage in KYSE30 cell. **c** GSDME expression profile in ESCC cell lines examined by western blot. **d** Western blot analysis of caspase3 and cleaved caspase3 after knocking down circPUM1 in KYSE30 cell. **e** Western blot analysis of pyroptosis-related proteins in KYSE30 cell after knocking down circPUM1. **f** Western blot analysis of the regulatory function of AMPK to the cleavage of GSDME following adding 2 μM AMPK inhibitor (Dorsomorphin dihydrochloride) in circPUM1 knockdown cell in KYSE30 cell and 200 μM AMPK activator (AICAR) in circPUM1 overexpressed cell in KYSE410 cell. **g** LDH release analysis after knockdown of circPUM1 and UQCRC2. **h** Immunofluorescence (IF) was performed to evaluate E-cadherin staining on KYSE30 cell after knocking down circPUM1, the scale bar is 30 μm

Interestingly, we found that circPUM1 was able to regulate pyroptosis, one of the cell death patterns. Following depletion of circPUM1 in KYSE30 cells, we observed that the morphological features of pyroptosis, which characterized by large bubbles blowing from the cell membrane (Fig. 6a). It has been well known that GSDMD or GSDME executes the pyroptosis process and their degraded N-terminal isotypes would assemble and induce cell membrane perforation in the end. As is shown in Fig. 6b, circPUM1 depletion increased the GSDME N-terminal expression with no impacts on the GSDMD N-terminal expression in ESCC cells. Hence, we inferred that GSDME participated in the pyroptosis process in ESCC cells. In view of this, we evaluated the expression of GSDME in ESCC cell lines simultaneously and found that many ESCC cell lines have varied expression of GSDME, indicating the involvement of pyroptosis in cell death for ESCC cells (Fig. 6c). In support of this observation, the cleaved caspase 3 increased significantly after the depletion of circPUM1 (Fig. 6d).

We also examined protein expressions related with pyroptosis signal pathway by western blot. Previous studies reported that AMPK pathway could induce caspase3/GSDME-mediated tumor cell pyroptosis.^29^ Furthermore, AMPK is activated by energy stress in response to low cellular ATP levels.^30^ In our study, circPUM1 regulates the mitochondrial energy metabolism. Given that, we detected whether circPUM1 could active AMPK pathway. As expected, silencing circPUM1 enhanced the phosphorylation of AMPK. Subsequently, circPUM1 knockdown in ESCC cells significantly decreased the total levels of caspase 3, while the expressions of cleaved caspase 3, cleaved caspase9, Cytc and Bax were notably increased (Fig. 6e). To determine the regulatory function of AMPK to the cleavage of GSDME, we added AMPK inhibitor (Dorsomorphin dihydrochloride) in circPUM1 knockdown cell in KYSE30 cell, and AMPK activator (AICAR) in circPUM1 overexpressed cell in KYSE410 cell. As is shown in Fig. 6f, decreased activity of AMPK inhibits the cleavage of GSDME, while enhanced activity of AMPK boosts the cleavage of GSDME. These results above demonstrate the involved regulatory role of AMPK to the cleavage of GSDME. Moreover, circPUM1 knockdown was seen to promote the release of lactate dehydrogenase, which is an indication of pyroptosis (Fig. 6g). Finally, circPUM1 knockdown made the cellular outline of ESCC cells change from spindle to spherical in the E-cadherin confocal immunofluorescence imaging assays (Fig. 6h). Taken together, circPUM1 may enhance the ESCC cell survival through regulating the pyroptosis mediated by activating GSDME.

## Discussion

Previous studies have demonstrated that tumor cells under survival pressure will undergo energy metabolism reprogramming and plays an important role in this process.^31,32^ In hypoxic microenvironment, accumulated HIF1α transcriptionally activates the expression of many genes which are involved in regulating the energy metabolism of tumor cells.^33,34^ However, the reprogramming of energy metabolism of tumor cells under hypoxia is an extremely complicated process. In this study, we demonstrated that circPUM1, a nuclear genome-derived circular RNA localized in mitochondria, responded to the induction of HIF1α protein level and increased following the accumulation of HIF1α in cells treated by CoCl_2_. Likely, circPUM1 acted as a scaffold protein for the assembly of mitochondrial complex III, and regulated oxidative phosphorylation of mitochondria through its interaction with UQCRC2. We also showed that circPUM1 might have oncogenic property as it promoted tumor growth probably via inhibiting pyroptosis of ESCC cells.

According to the previous findings, circRNAs usually localize in the cytoplasm and mainly plays roles as endogenous competitive RNA of microRNA.^35^ However, through RNA pull-down assay and mass spectrometry, we made the first demonstration that circPUM1 was co-localized with mitochondrial complex III core protein UQCRC2 in mitochondria. As a semi-autonomous organelle, the function of mitochondria is regulated by both nuclear genome and mitochondrial genome.^36,37^ Recently, it has been shown that circRNA SCAR derived from mitochondria could regulate the switch of mitochondrial permeability transition pore.^38^ In our study, we confirmed that nuclear genome-derived circPUM1 could enter the mitochondria, directly interact with critical mitochondrial proteins and regulate the oxidative phosphorylation of mitochondria. Together with the reports by others, this finding supports that circRNAs may play important roles in mitochondria.

It has been well accepted that the tumor cells were always in hypoxia as a result of rapid proliferation.^39,40^ In hypoxic microenvironment, the accumulation of HIF1α can promote glycolysis of tumor cells and maintain the survival of tumor cells.^41,42^ Interestingly, the oxidative phosphorylation of mitochondria is not inhibited, but rather augmented in in some cancers.^10^ However, it remains ambiguous how the oxidative phosphorylation of tumor cells is enhanced. Our studies suggested that circPUM1, which is located in mitochondria, could regulate the mitochondrial membrane potential by interacting with UQCRC2. Indeed, the seahorse assay indicated that circPUM1 could promote the oxidative phosphorylation of mitochondria. These observations largely explained the mechanism of enhanced oxidative phosphorylation in tumor cells. Additionally, the expression level of circPUM1 was positively correlated with the protein level of HIF1α, indicating that HIF1α was also indirectly involved in the regulation of oxidative phosphorylation of mitochondria in ESCC cells. We speculated that the oxygen consumption of ESCC cells increased through enhanced oxidative phosphorylation, which further leads to hypoxia and followed by induction of HIF1 α as a positive feedback loop. Therefore, our findings further enrich the role of HIF1α in the process of energy metabolism of tumor cells. HIF1α may participate in the regulation of glycolysis and oxidative phosphorylation during the tumor progression.

Pyroptosis is a newly discovered programmed cell death which is closely related to inflammation and tumorigenesis.^43,44^ Previous reports suggest that the decrease of mitochondrial membrane potential could lead to the augment of mitochondrial ROS.^45^ Mitochondria-derived ROS is the major source of intracellular ROS. It has been found that the increasing intracellular ROS and cleaved caspase-3 protein can promote pyroptosis of tumor cells by activated GSDME.^46^ Bo Zhou et al reported that mitochondrial membrane protein Tom20 could sense iron-activated ROS signaling and enhance pyroptosis in melanoma cells.^47^ In our study, we found that mitochondria-located circPUM1 was able to inhibit pyroptosis by modulating intracellular ATP level. Our study further confirms the important role of mitochondria in pyroptosis. Moreover, these findings suggest circPUM1 as a novel target molecule for the development of therapeutic approaches in ESCC tumors.

## Materials and methods

### Cell lines and cell culture

The YES2, KYSE30, KYSE70, KYSE140, KYSE150, KYSE180, KYSE410, KYSE450, KYSE510, COLO680N cells were gifts from Professor Yutaka Shimada (Kyoto University). All the ESCC cell lines were cultured in RPMI1640 (Gibco) with 10% fetal bovine serum at 37 °C with 5% CO_2_. The immortalized esophageal epithelium cell line NE2 cell was gift from Professor Enmin Li (Shantou University), and was cultured in a 1:1 mixture of EpiLife and dKSFM (Gibco) at 37 °C with 5% CO_2_.

### RNA interference

For gene knockdown experiments, 1.5 × 10^5^ KYSE30 cell were seeded on six-well culture plates and transfected with oligonucleotides using lipofectamine 2000 according to the manufacturer’s instructions. And the transfected cells were cultured at 37 °C with 5% CO_2_ for 48 h. For stable expression, the pLKO.1 lentiviral vector containing the desired gene, together with the packing plasmid psPAX2 and envelop plasmid pMD2. G, were transfected into 293T cells at a ratio of 4:3:1. And 48 h later, the cell supernatants containing lentiviral particles were collected to infect KYSE30 for another 24 h, and then 1 μg/ml puromycin was added to select the positive cells. Detailed sequence information is listed in additional file 1: Supplementary Table S1.

### Intracellular hypoxic model construction

1.5 × 10^5^ KYSE30 cell was seeded in six-well culture plate, and CoCl_2_ (Sigma-Aldrich) was added to cells to the final concentration 200 μM. Samples were collected at 6 h, 12 h, 18 h, 24 h, 30 h time points. Western blot was performed to examine the dynamic change of HIF1α.

### Western blot

Total cellular lysates were prepared in lysis buffer. Identical quantities of proteins were separated by SDS-PAGE and then transferred onto PVDF membranes. After incubation with corresponding antibodies, the membranes were incubated with horseradish peroxidase (HRP)-conjugated anti-rabbit IgG or anti-mouse IgG secondary antibody, and bands were detected using Amersham Imager 600 (GE, USA). β-actin was used as a loading control. The information of antibodies is described in additional file 2: Supplementary Table S2.

### Whole transcriptome sequencing and RT-qPCR

Extracting RNA after cell exposing to 200 μM CoCl_2_ at the time point of 18 h. And then, the RNA was used to performed the whole transcriptome sequencing in NovelBio Cloud Analysis platform (Shanghai). RT-qPCR was used to verify the changed circular RNA by using Premix Ex Taq kit (Takara) and a CFX96 Touch real-time PCR system (BIO-RAD). We design primers that covered the junction sites for circPUM1, and the target gene level was normalized to β-Actin. The information of primers is described in additional file 3: Supplementary Table S3.

### Cytosolic/nuclear fractionation

The KYSE30 cells were harvested and washed with pre-cold phosphate-buffered saline (PBS), and then cells were centrifuged at 500 × *g* for 5 min. After removal of the residue PBS, the cell pellet was resuspended with 100 μL nuclear and cytoplasmic extraction reagent (140 mM NaCl, 1.5 mM MgCl_2_,10 mM Tris-HCl pH = 8.5, 0.5% NP40), and then the mix were incubated on ice for 5 min, and centrifuged at 5000 × *g* for 5 min. Then remove the supernatant to a new tube, and 1 ml Trizol regent was added. The remained nuclear pellet was also resuspended in 1 ml Trizol reagent after a wash with PBS. RNA in each fraction was extracted and subjected to RT-qPCR analysis to detect the level of nuclear control transcript (NEAT1), cytoplasmic control transcript (GAPDH) and circPUM1.

### Northern blot

20 μg of total RNA was mixed into the 2 × loading buffer, and then RNA was separated on 1% denaturing agarose gels before transferring to Hybond-N (GE). Membranes were then dried and crosslinked at 120 °C for 30 min. Digoxin-labeled DNA probes (Supplementary Table S1) were incubated with membranes overnight at 55 °C before visualizing bands using the ImageQuant LAS 4000 (GE, USA). The probes used for Northern Blot are listed in additional file 1: Supplementary Table S1.

### Proliferation assay

The proliferation ability of KYSE30 and KYSE410 after transfecting with siRNAs and circPUM1 overexpression plasmid was monitored by using the xCELLigence Real-Time Cell Analyzer (RTCA)-MP system (Acea Biosciences/ Roche Applied Science). Transfected cell with siRNAs in six-well culture plate for 24 h, and circPUM1 overexpression plasmid for 48 h, then 50 μL culture medium was added into each well of E-Plate 96 (Roche Applied Science) to obtain equilibrium, and then 3000 cells in 100 μL culture medium were seeded in E-Plate96, which was subsequently locked in RTCA-MP device at 37 °C with 5% CO_2_. Cell index that reflects cellular proliferation on biocompatible microelectrode coated surfaces was read every 15 min automatically and the recorded curve was shown as cell index±s.e.m.

### Colony formation assay

1000 transfected cells were seeded into six-well culture plate and incubated at 37 °C with 5% CO_2_ for about 10 days. And after a wash with pre-cold PBS, cells were fixed with methanol for 20 min and stained with crystal violet for 15 min. Colonies were examined and calculated by G: box (Syngene) automatically.

### RNA in situ hybridization (ISH) assay, confocal assay and High-resolution imaging

To examine the localization of circPUM1, cells were seeded in glass-bottom cell culture dishes (NEST) to a confluency of approximately 40–60%. Mitotracker was added to the cell culture medium for 2 h. And after that FISH analysis was then performed using a fluorescence in situ hybridization kit (Ruibobio). Briefly, cells were fixed with 4% paraformaldehyde for 10 min at room temperature and then washed 3 times with PBS, and 1% Triton-100 was used to penetrate the cells for 5 min at 4 °C. Pre-hybrid solution was used to block cell at 37 °C for 30 min to reduce the background signal. Then the hybridization solution containing the circPUM1 probe (Supplementary Table S1) was added to the cells, and the cells were incubated overnight at 42 °C in the dark. The cells were then washed separately with 4 × SSC, 2 × SSC and 1 × SSC. Finally, view the cells in the Ultraview VoX system (PE, Germany). For the confocal assay, after staining the probe, the cells were incubated with the antibody overnight at 4 °C. The cells were then incubated with Alexa Fluor 488-labeled for 2 h at room temperature in the dark. Images were then captured and visualized by the Ultraview VoX system (PE, Germany). For super-resolution microscopy of mitochondria, live cell that transfected with si-circPUM1 and si-con were used, and images were captured by DeltaVision OMX SR (GE, USA). The probes used for ISH are listed in additional file 1: Supplementary Table S1.

### Tumor xenograft model

1 × 10^6^ cells (shNC, shcircPUM1-1, and shcircPUM1-2) were inject into the right and left backside region of 4-week-old male athymic nude mice, respectively. Tumor size was measured using calliper. Tumor volume was calculated by using the formula (L × W^2^)/2, where L is the length of tumor while W is the width of the tumor. All animal experiments were approved by the Institutional Animal Care and Use Committee of Peking University Cancer Hospital & Institute.

### RNA pulldown and mass spectrometry assay

Transcripted different length of sense probes and anti-sense probes (Supplementary Table S3), which had a T7 promoter ahead of the sequences (Thermo Fisher Scientific). And then, transcription products were signed a biotin at 3’ end of these probes according to the manufacturer’s instructions (Thermo Fisher Scientific). For RNA pulldown assay, firstly, the labeled probes were denatured at 95 °C for 2 min and incubated on ice immediately, then Streptomycin beads was blocked by using tRNA and 20% BSA, and at the same time, cell lysates were pre-cleared by using streptomycin beads. At the next step, probes, pre-cleared cell lysates and blocked streptomycin beads were mixed together. Then, wash buffer containing 150 mM NaCl was used to wash this complex for 6 times and got proteins that could bind with probes eventually. Silver dye was used to reflect the binding ability of proteins with sense probes and anti-sense probes. We choose the suitable bands, which were thicker and had the same trends in all different anti-sense probe groups compared with the sense probe groups to perform Mass spectrometry assay by using Q Exactive (Thermo Fisher Scientific) at the CapitalBio Technology Inc. Primers for in vitro transcription is described in additional file 3: Supplementary Table S3.

### Interaction prediction

To determine whether circPUM1 interacts with UQCRC2, we used the protein-RNA docking analysis tool NPDock Server that generated 20,000 models to identify the best model. NPDock Server combines GRAMM for docking, scoring, clustering and refining the best scored docked complex in the end.

### Mitochondria isolation

Intact mitochondria were isolated by using mitochondria isolation kit according to the manufacturer’s instructions (Invent). In brief, 5–40 × 10^6^ cells were collected by low speed centrifugation, and 250 μL Buffer A was added to cells and incubated on ice for 10 min. Transferred the mix to the filter cartridge and centrifuge at 16,000 × *g* for 30 s. Resuspended the pellet and centrifuged at 3000 rpm for 1 min. Transferred the supernatant to a new tube and added 400 μL Buffer B to the same tube, then centrifuged the tube at 16,000 × *g* for 10 min. Removed the supernatant completely and resuspended the pellet in 200 μL Buffer B, and centrifuged the tube at 16,000 × *g* for 5 min. Transferred the supernatant to a new tube, and added 1.6 ml pre-cold PBS to the tube and centrifuged at 16,000 × *g* for 15 min, and obtained the mitochondria at the bottom of the tube in the end.

### RNA Binding Protein Immunoprecipitation (RIP)

RIP assay was performed to examine the interaction between UQCRC2 and circPUM1 according to the manufacture’s instruction (MILIpore). Briefly, lysed the mitochondria isolated by mitochondria isolation kit with lysis buffer, immunoprecipitated with Anti-UQCRC2 to UQCRC2 with protein A/G magnetic beads, immobilized magnetic beads bound complexes with magnet and wash off unbound materials, and extracted RNA for analysis.

### Cellular oxygen content test assay

1.5 × 10^5^ KYSE30 cell were seeded in six-well culture plate. When cells had been transfected with siRNAs, plasmids for 48 h or exposed to CoCl_2_ for 18 h, Hypoxia Green reagent (Thermo Fisher Scientific) was added to cells to the final concentration of 1 mM. Incubated the cells at 37 °C for 2–3 h, and then washed cells once in PBS, and BD LSRII flow cytometer was used to analyze the stained cells, and the data were processed using FlowJo software.

### Mitochondrial membrane potential assay

1.5 × 10^5^ KYSE30 or 1.5 × 10^5^ KYSE410 cells were seeded in glass-bottom cell culture dish (NEST), after cells were transfected with siRNA or plasmids for 48 h, Jc-1 working solution (Bryotime) was used to stain cells at 37 °C for 20 min, and then pre-cold washing solution was used to wash cells twice before capturing images by using Ultraview VoX system.

### ROS and MitoSOX generation assay

1.5 × 10^5^ KYSE30 or 1.5 × 10^5^ KYSE410 cells were seeded in six-well culture plate. When cells had been transfected with siRNAs or plasmids for 48 h, DCFH-DA (Bryotime) or MitoSOX (Thermo Fisher Scientific) working solution were added to cells for the final concentration of 5 μM at 37 °C for 20 min. Fresh medium was used to wash cell 3 times. Cells were collected and used for flow cytometry analysis afterward.

### OCR and ECAR rate measurements

Assays were performed using the Seahorse XF 96 analyzers (Seahorse Bioscience, Agilent). OCR measurements were performed by using Agilent Seahorse XF Mito Stress Test Kit (103015-100) according to the manufacturer’s instruction. Briefly, 8 × 10^3^ cells/well were seeded in a 96-well XF cell culture microplate in growth medium 24 h before assay. Hydrate a sensor cartridge in a Seahorse XF Calibrant at 37 °C in a non-CO_2_ incubator overnight. And then OCR was measured with an XF96 analyzer in XF base medium (pH 7.4) containing 1 mM pyruvate, 2 mM glutamine, and 10 mM glucose following sequential addition of Oligomycin (4 μM), FCCP (1 μM) and antimycin A (0.5 μM). Data were analyzed by the Seahorse XF Mito Stress Test Reporter Generator package. ECAR measurements were performed by using Agilent Seahorse XF Mito Stress Test Kit (103020-100) according to the manufacturer’s instruction. The process of ECAR measurements was almost the same with OCR measurements, except the sequential addition of Glucose (10 mM), Oligomycin (1 μM), 2-DG (50 mM).

### ATP generation assay

1.5 × 10^5^ KYSE30 or 1.5 × 10^5^ KYSE410 cells were seeded in six-well culture plate. 200 μL lysis buffer was added to cells after cells were treated with siRNA or plasmids for 48 h. 100 μL assay buffer was added to the test tubes for 3–5 min to decrease the background. Then 20 μL cell lysate was added to test tubes, and chemiluminescence absorbance was calculated to obtain the concentration of ATP in different cells (Byrotime).

### LDH release assay

LDH release assay was performed to test the cell vitality after knockdown of circPUM1 and UQCRC2 according to the manufacture’s instruction. Briefly, 1.5 × 10^5^ KYSE30 cells were seeded in six-well culture plate. Collect the cellular supernatant after cells were treated with siRNAs for 48 h. Add 2–50 μL samples into duplicate wells of a 96-well plate. Bring samples to a final volume of 50 μL with LDH assay buffer. Add 50 μL of the master reaction mix to each of the wells, and take the initial measurement at 450 nm and noted as (A_450_) _initial_. Incubate the plate at 37 °C taking measurements every 5 min until the value of the most active sample is greater than the value of the highest standard. The final measurement (A_450_) _final_ would be penultimate reading or the value before the most active sample. The time of the penultimate reading is marked as T _final_. The LDH activity of a sample may be determined by equation described in the manufacture’s instruction (Sigma).

### Statistical analysis

IBM SPSS version 25 software packages was used to assess the differences between experimental groups. Data were analyzed by two-tailed Student’s *t* test. All data presented in this study are means ± SEM. In all experiments, differences were considered to be significant when *P* was less than 0.05. *P < 0.05, **P < 0.01, ***P < 0.001. All vitro assays were repeated at least 3 times.

## Supplementary information

Supplementary Materials

## Data Availability

Further information and requests for resources and reagents should be directed to and will be fulfilled by the corresponding author, Q.Z. (zhanqimin@bjmu.edu.cn).
