# Supplementary material for: Nuclear genome-derived circular RNA circPUM1 localizes in mitochondria and regulates oxidative phosphorylation in esophageal squamous cell carcinoma
Source: Signal Transduct Target Ther. 2022 Feb 14;7:40. doi: 10.1038/s41392-021-00865-0 (PMC8841503; doi:10.1038/s41392-021-00865-0)
Supplement: Supplementary file 1 — Supplementary Materials [file 41392_2021_865_MOESM1_ESM.docx]

Supplementary Materials for

**Nuclear genome-derived** **circular RNA circPUM1 localizes in** **mitochondria and regulates oxidative phosphorylation in esophageal squamous cell carcinoma**

Wei Gong^1,§,^ Jiancheng Xu^2, §^, Yan Wang^1^, Qingjie Min^1^, Xu Chen^1^, Weimin Zhang^1^, Jie Chen^1^, Qimin Zhan^1,3,^,^4*^

Correspondence to: zhanqimin@bjmu.edu.cn

**This PDF file includes:**

Supplementary Figures. S1 to S5

Supplementary Tables S1 to S3


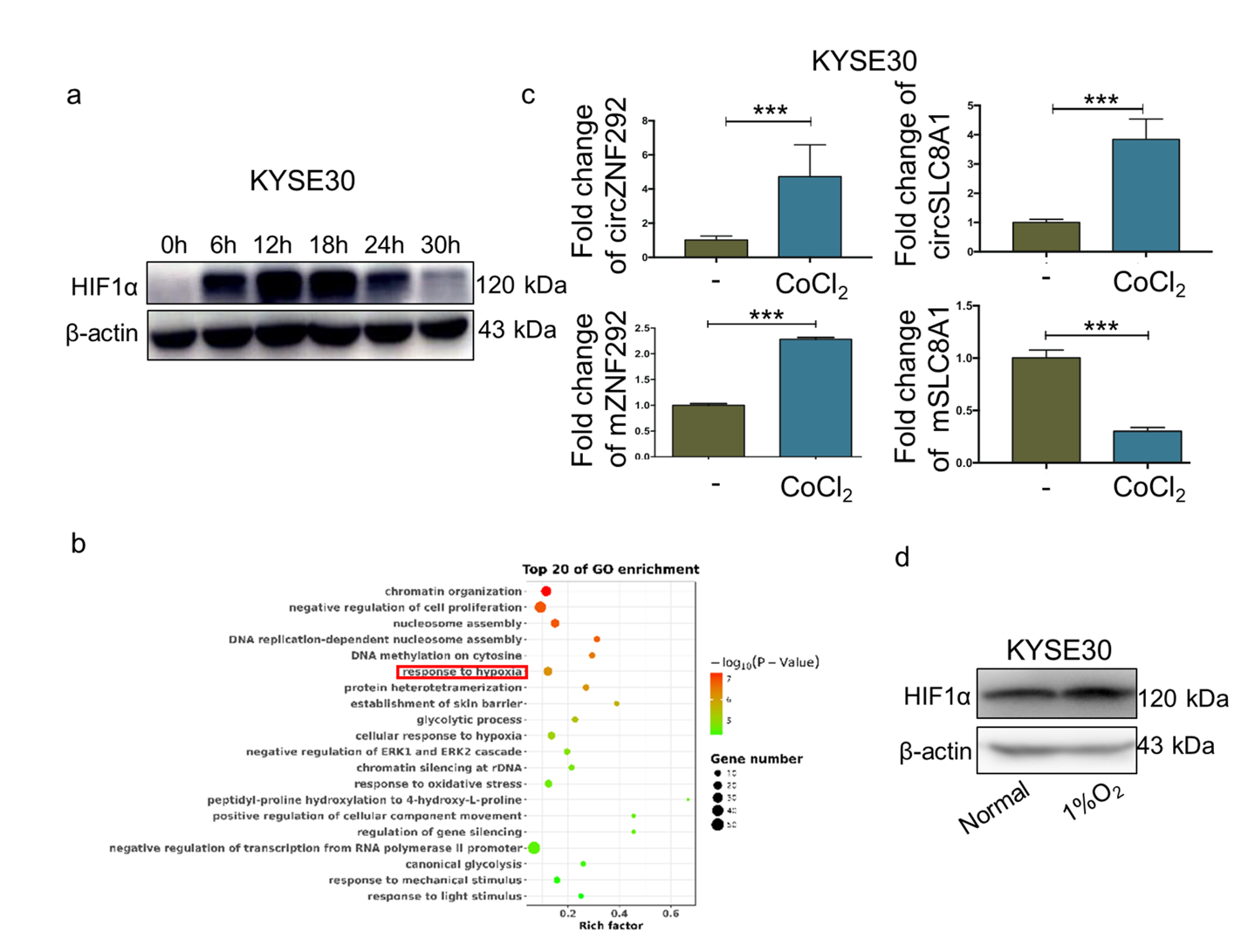


**Supplementary Fig. S1. HIF1α induces expression of circPUM1.**

(a) Western blot analysis of the dynamic change of HIF1α in different time points when KYSE30 cell was treated with 200 μM CoCl_2_.

(b) Gene Ontology enrichment analysis of differential expressed genes.

(c) RT-qPCR assay to determine circZNF292, mZNF292, circSLC8A1 and mSLC8A1 expression level when KYSE30 cell was treated with 200 μM CoCl_2_ for 18 h.

(d) Western blot analysis of the change of HIF1α when KYSE30 cells were incubated in 1% oxygen for 24 h.

**
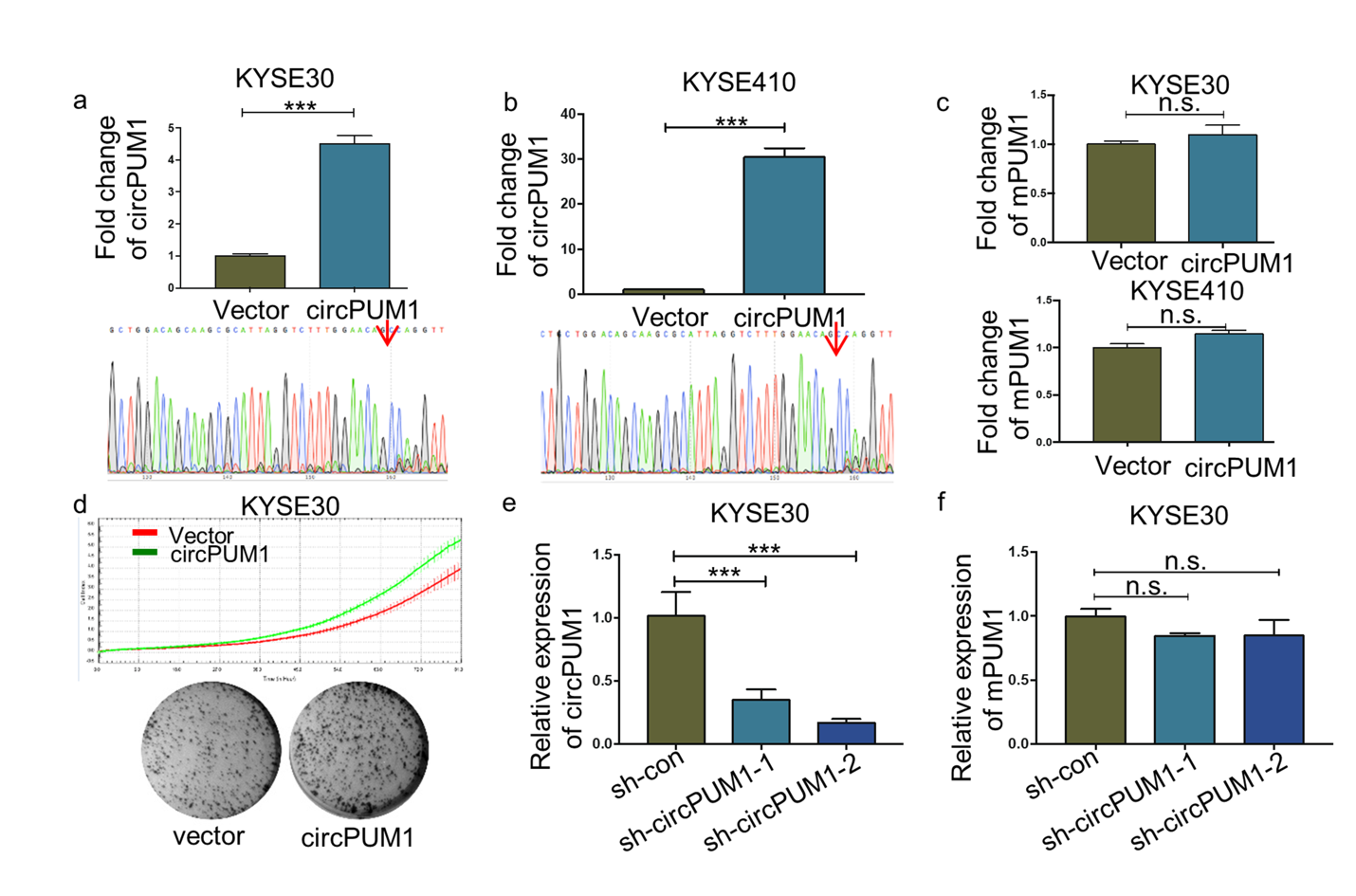
**

**Supplementary Fig. S2. knock-down or over-expressed circPUM1 will not interfere the expression of PUM1 message RNA.**

(a) RT-qPCR analysis for the fold change of circPUM1 in KYSE30 that transfected with the circPUM1-overexpressed plasmid and the blank control vector, and the following Sanger sequencing.

(b) RT-qPCR analysis for the fold change of circPUM1 in KYSE410 that transfected with the circPUM1-overexpressed plasmid and the blank control vector, and the following Sanger sequencing.

(c) RT-qPCR analysis for the fold change of mPUM1 after overexpressing circPUM1 in KYSE30 cell (upper) and KYSE410 cell (lower).

(d) The growth curve that monitored by the RTCA-MP system and colony formation analyses following the overexpression of circPUM1 in KYSE30 cell.

(e) RT-qPCR analysis of the relative expression of circPUM1 in the stable circPUM1 knockdown KYSE30 cell.

(f) RT-qPCR analysis of the relative expression of mPUM1 in the stable circPUM1 knockdown KYSE30 cell.


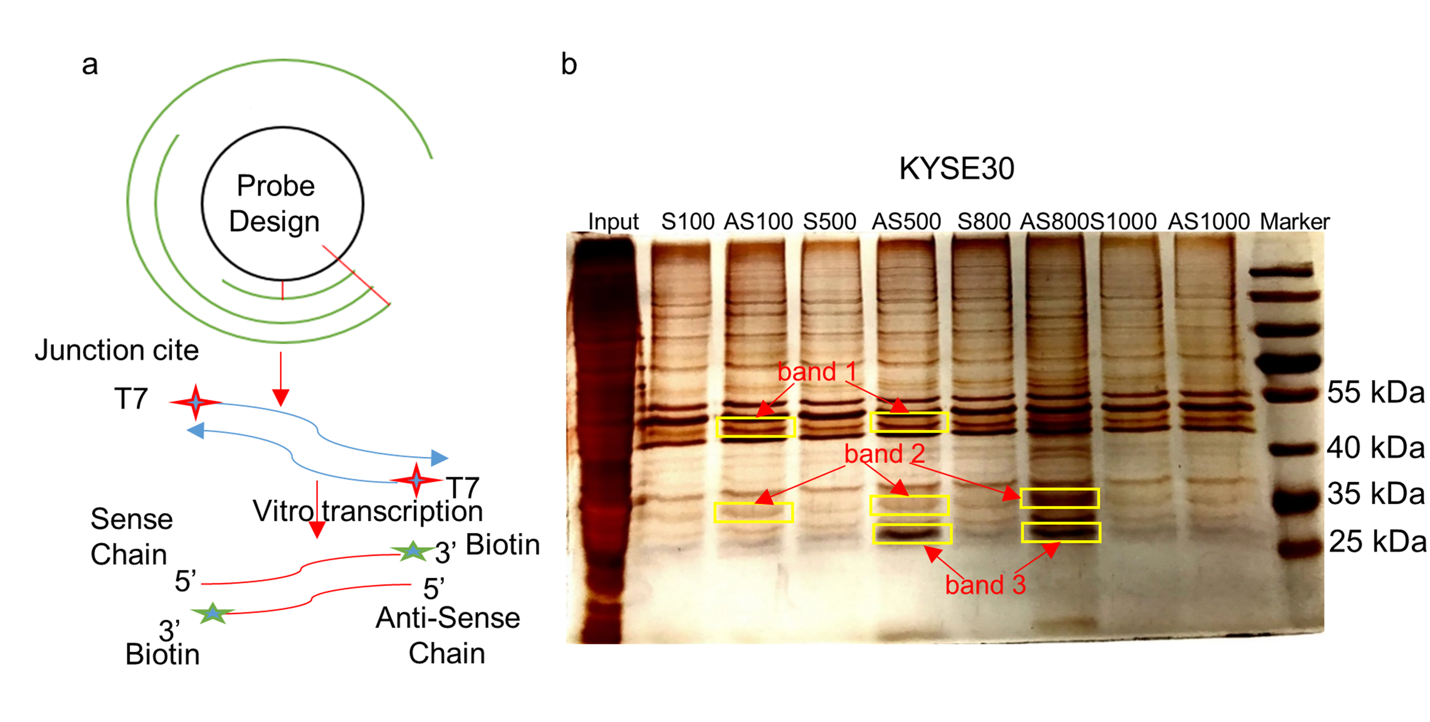


**Supplementary Fig. S3. Probe design strategy and MASS spectrometry.**

(a) Graphical summary of probes design strategy.

(b) Silver staining of the precipitated proteins pulled down by different length of sense probe and anti-sense probes in KYSE30 cell (frame, protein band for Mass spectrometry).

**
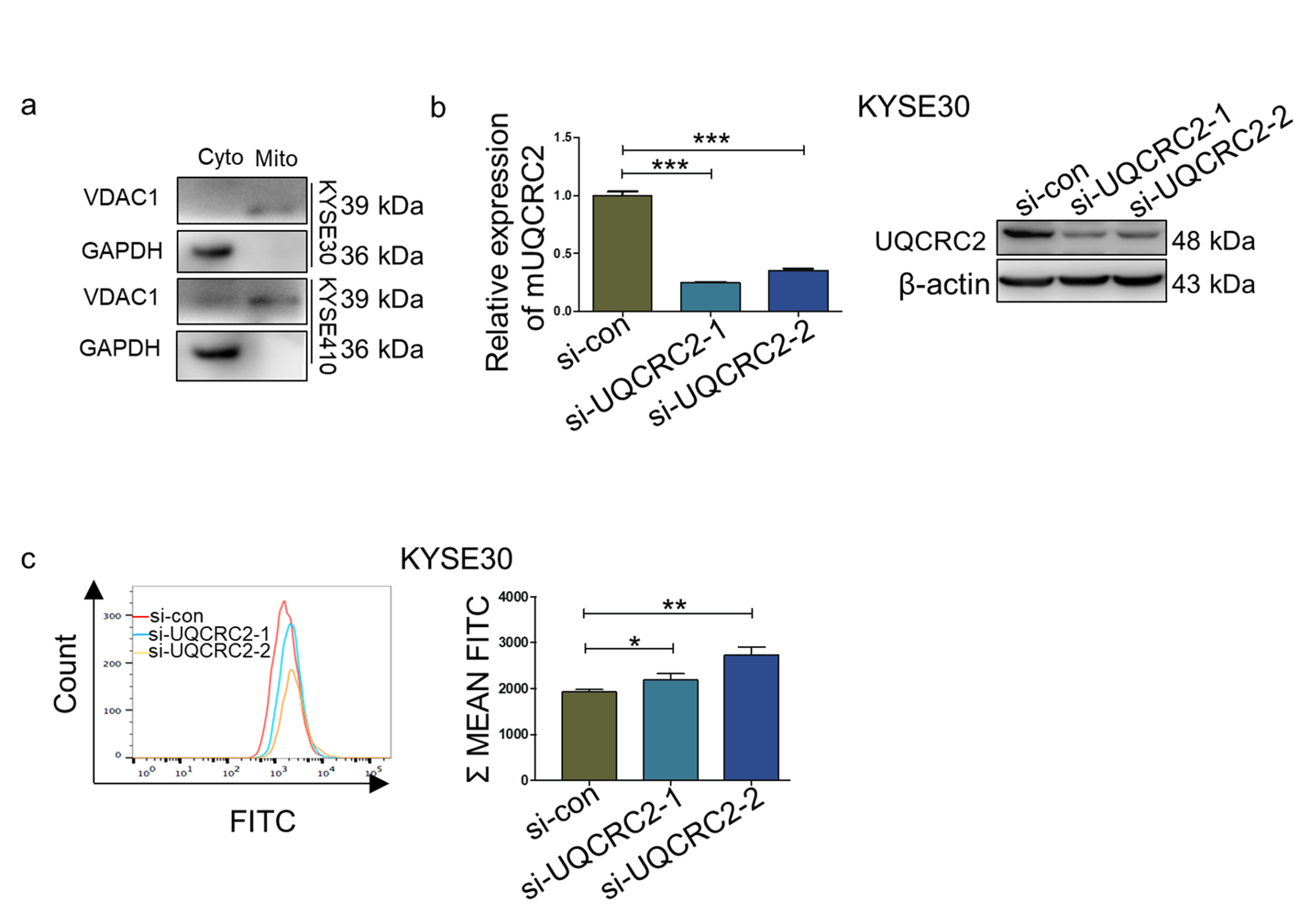
**

**Supplementary Fig. S4. knock-down UQCRC2 induces low oxygen concentration in ESCC cells.** (a) Western blot was used to test the purity of isolated mitochondria in KYSE30 and KYSE410 cells, VDAC1 was used as a mitochondrial specific marker, while GAPDH was used as a cytoplasmic marker.

(b) RT-qPCR and western blot analyses for the knockdown efficiency of si-UQCRC2 in KYSE30 cell.

(c) Flow cytometry was performed to test hypoxic cells after knocking down UQCRC2.


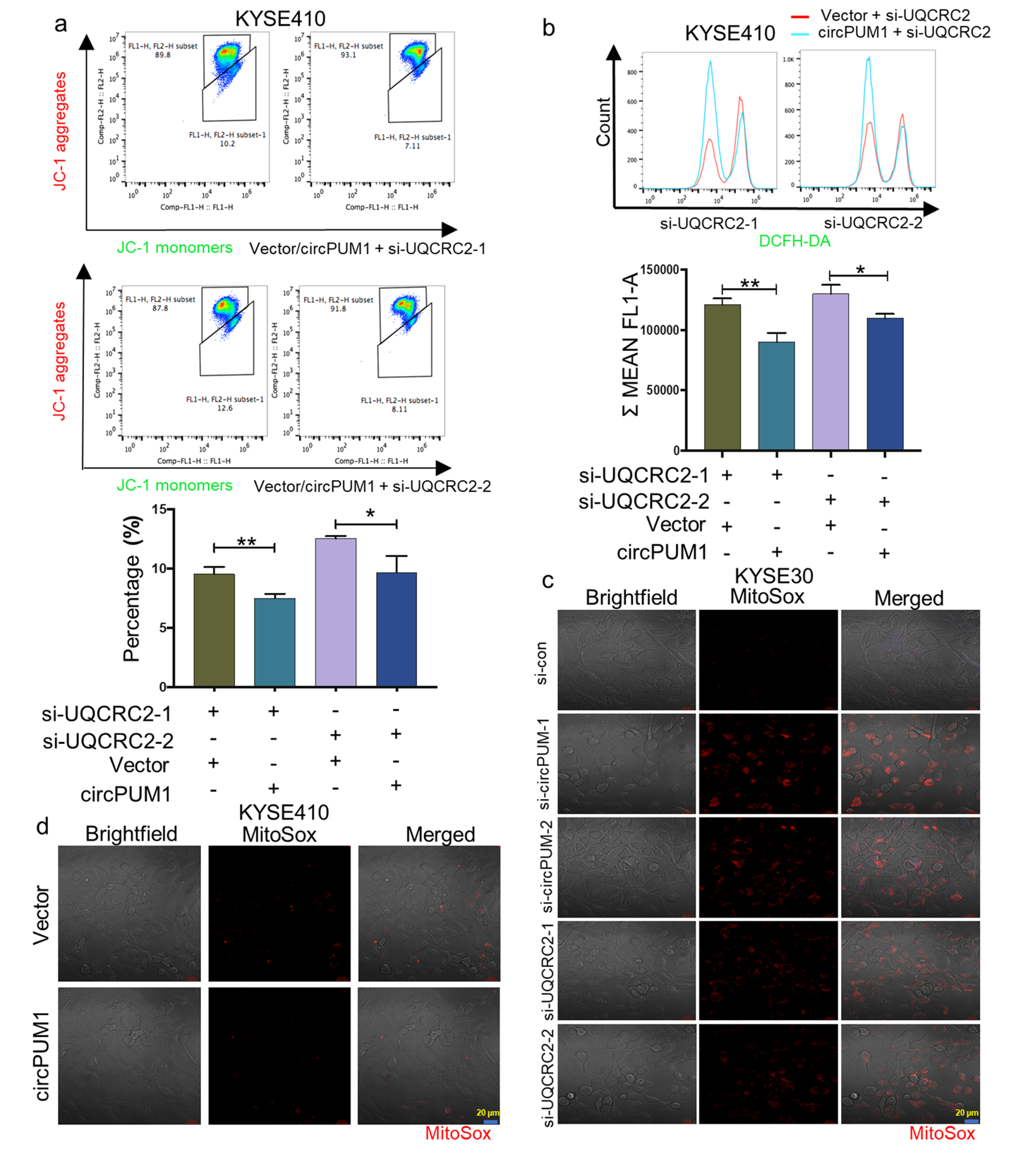


**Supplementary Fig. S5. CircPUM1 regulates MMP, ROS, MitoSox.**

(a) Rescue experiment was performed to analyze the change of MMP after overexpressing vector and circPUM1 in KYSE410 cells, which were transfected with the si-UQCRC2 24 h before.

(b) Rescue experiment was performed to analyze the change of ROS generation after overexpressing vector and circPUM1 in KYSE410 cells, which were transfected with the si-UQCRC2 24 h before.

(c) Fluorescence microscope was used to observe the MitoSox generation after knocking down circPUM1 and UQCRC2 in KYSE30 cells, the scale bar is 20 μm.

(d) Fluorescence microscope was used to observe the MitoSox generation after overexpressing circPUM1 in KYSE410 cells, the scale bar is 20 μm.

Graphical Abstract


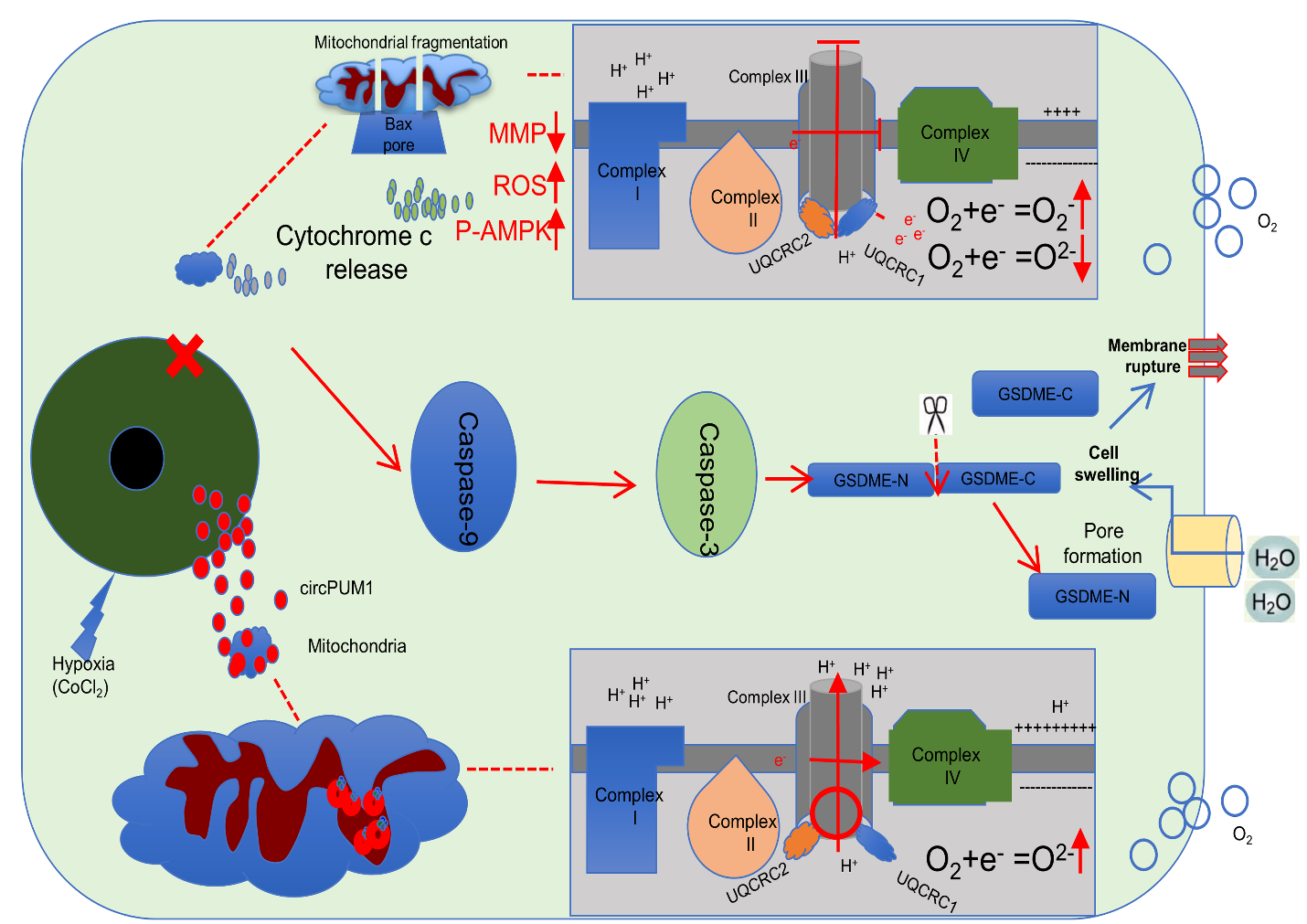


Table S1.

siRNAs, Digoxin labeled probes, Alexa 488 labeled probes

| siRNA sequences Target sequence (5’-3’) | |
| --- | --- |
| si-cPUM1-1 | CTTAAACCTGGCTGTTCCA |
| si-cPUM1-2 | AAACCTGGCTGTTCCAAAG |
| si-HIF1α-1 | GAAGGAACCTGATGCTTTA |
| si-HIF1α-2 | GCCACTTCGAAGTAGTGCT |
| si-UQCRC2-1 | GCACCAGAATTTCGTCGTT |
| si-UQCRC2-2 | GCTGAAAGCTGGATACCTA |
| Digoxin labeled probes Sequence (5’-3’) | |
| circPUM1 | TTGGAACAGCCAGGTTTAAGCTTGA |
| Alexa 488 labeled probes Sequence (5’-3’) | |
| circPUM1 | TTGGAACAGCCAGGTTTAAGCTTGA |

Table S2.

information of antibodies

| Antibodies |  | | IF | Western blot | | RIP | | Co-IP |
| --- | --- | --- | --- | --- | --- | --- | --- | --- |
| HIF1α | | Abcam Cat#ab2185 |  | 1:1000 | |  | |  |
| UQCRC2 | | Abcam Cat#ab203832 |  | 1:1000 | | 2μg | | 2μg |
| GSDME | | Abcam Cat #ab215191 |  | 1:1000 | |  | |  |
| Cyto c | | CST Cat #11940 |  | 1:1000 | |  | |  |
| Bax | | CST Cat #2772 |  | 1:1000 | |  | |  |
| Caspase3 | | CST Cat #9662 |  | 1:1000 | |  | |  |
| Cleaved Caspase3 | | CST Cat #9661 |  | 1:1000 | |  | |  |
| Cleaved Caspase9 | | CST Cat #9509 |  | 1:1000 | |  | |  |
| GSDMD | | CST Cat #93709 |  | 1:1000 | |  | |  |
| Cleaved GSDMD | | CST Cat #36425 |  | 1:1000 | |  | |  |
| AMPKα | | CST Cat#5831 |  | 1:1000 | |  | |  |
| Phospho-AMPKα (Thr172) | | CST Cat#2535 |  | 1:1000 | |  | |  |
| β-Actin | | Santa Cruz Biotechnology Cat#sc-8432 |  | 1:2000 | |  | |  |
| E-cadherin | | Santa Cruz Biotechnology Cat #sc-7870 | 1:50 | 1:1000 | |  | |  |
| UQCRC1 | | PROTEINTECH Cat #21705-1-AP |  | 1:500 | |  | | 2μg |
| Anti-Mouse IgG, HRP | | Promega Corporation Cat #W4021 |  | 1:5000 | |  | |  |
| Anti-Rabbit IgG, HRP | | Promega Corporation Cat #W4011 |  |  | |  | |  |
| AMCA– conjugated Anti-Rabbit IgG | | CST Cat #SA00010-2 | 1:200 | |  | |  |  |
| IgG/Alexa Fluor 488 | | ZSGB-BIO Cat #ZF-0511 | 1:200 | |  |  | |  |

**Table S3**

The list of primers

| Primers used in RT-qPCR | | | | | |
| --- | --- | --- | --- | --- | --- |
| Name | Sequence (5’-3’) | | | | |
|  | Forward | | Reverse | | |
| circPUM1 | AGCTTAAACCTGGCTGTTCCA | | | GGAGCAGCGCTGATGATGTA | |
| HIF1α | CACCACAGGACAGTACAGGAT | | CGTGCTGAATAATACCACTCACA | | |
| UQCRC2 | TTCAGCAATTTAGGAACCACCC | | GGTCACACTTAATTTGCCACCAA | | |
| β-actin | TGGCACCCAGCACAATGAAG | | AAGCATTTGCGGTGGACGAT | | |
| mPUM1 | TGAATCCAGCAAAGATGGACC | | ATACTCCACCATGCTAACGCC | | |
| Primers for in vitro transcription | | | | | |
| Name | | Sequence (5’-3’) | | | |
|  |  | Forward | | | Reverse |
| T7-cPUM1-sense-100 | | taatacgactcactataggCTGTTCCAAAGACCTAATGC | | | CTAAACCGATGTGCGGCTGATGAGC |
| T7-cPUM1-sense-500 | | taatacgactcactataggGTCCAGTTCTTTCTACGGCAA | | | CTAAACCGATGTGCGGCTGATGAGC |
| T7-cPUM1-sense-800 | | taatacgactcactataggGGGTCTGGCAGCAGGCATGCC | | | CTAAACCGATGTGCGGCTGATGAGC |
| T7-cPUM1-sense-1000 | | taatacgactcactataggGAGCAGCAAGCTGCCGCTGCC | | | CTAAACCGATGTGCGGCTGATGAGC |
| T7-cPUM1-Anti-sense-100 | | taatacgactcactataggACTAAACCGATGTGCGGCTGA | | | CTGTTCCAAAGACCTAATGC |
| T7-cPUM1-Anti-sense-500 | | taatacgactcactataggACTAAACCGATGTGCGGCTGA | | | GTCCAGTTCTTTCTACGGCAA |
| T7-cPUM1-Anti-sense-800 | | taatacgactcactataggCTAAACCGATGTGCGGCTGA | | | GGGTCTGGCAGCAGGCATGCC |
| T7-cPUM1-Anti-sense-1000 | | taatacgactcactataggACTAAACCGATGTGCGGCTGA | | | GAGCAGCAAGCTGCCGCTGCC |
